# Predictors of Return to Work Among Patients Attending a Long-term Treatment and Rehabilitation Service for Functional Neurological Disorder (FND) and Related Conditions

**DOI:** 10.1192/j.eurpsy.2023.2191

**Published:** 2023-07-19

**Authors:** M. Gheis, S. Ellis, K. Lenk, C. Lamb

**Affiliations:** 1 Psychiatry, University of British Columbia; 2 Neurorehabilitation, Vancouver Island Health Authority; 3 Adminstration, Program for Functional Neurological Disorder; 4 Clinical physiotherapy, Dockside Community Rehabilitation, Victoria, Canada

## Abstract

**Introduction:**

Limited data is available on the prognosis of patients with FND concerning their ability to return to work.

**Objectives:**

To identify factors associated with the ability to return to work in patients with FND following treatment and rehabilitation.

**Methods:**

We retrospectively assessed the employment outcomes of 79 consecutively evaluated patients. Patients were referred at the inception of an FND Program for adults. The majority of patients were unemployed, on sickness leave and or disability benefits at the time of their referral (n=71). Their median age was 48 years. Most patients were of female gender (*n*=50), in a relationship (*n*=53), with no dependants (*n=*64). Most patients had a referral diagnosis of mixed functional neurological symptoms (*n*=35), presenting with a combination of motor, sensory, cogniform or dissociative seizure symptoms. Among patients distinct phenomenological presentations, the most common referral diagnosis was functional sensory disorder (*n*=16). Twenty two patients had a concurrent structural neurological disorder. Seven patients had an accident compensation claim, and twenty had a workers’ compensation or employment insurance claim at the time of referral.

**Results:**

Approximately 30 % of patients were able to return to some work (*n*=24) within five years or less, and all those who were in employment at the time of the referral continued to hold a job for the duration of their treatment. We identified a negative correlation between patients’ ability to return to work and the length of employment interruption, with patients more recently out of work (within a year prior to the referral) being most able to return to work (odds ratio = 2; 95% CI, 1.2 to 3.8). We previously analyzed employment figures at 18 months of the service operation. Return to work was moderately lower at that point at 19%, but with maintained negative correlation with the length of employment interruption.

There was a negative correlation between having a work-related financial claim and the ability to return to work (*p* <0.001). There was no statistically significant correlation between demographic variables (gender, age, relationship status, or having dependants) and the ability to return to work, nor was there a statistically significant correlation between the phenomenology of Functional Neurological Disorder (motor, sensory, cogniform, non-epileptic attack disorder or mixed) and the ability to return to work.

**Conclusions:**

Early and continuous treatment of employed or recently unemployed patients with Functional Neurological Disorder is associated with better occupational outcomes. Having a work-related compensation claim is correlated with negative occupational outcomes. There is a need for further research into occupational rehabilitation, specially for patients receiving work-related compensation claim.

**Disclosure of Interest:**

None Declared

